# Quarantine, physical distancing and social isolation measures in individuals potentially exposed to SARS-CoV-2 in community settings and health services: a scoping review

**DOI:** 10.3126/nje.v12i2.43838

**Published:** 2022-06-30

**Authors:** Tereza Brenda Clementino de Freitas, Rafaella Cristina Tavares Belo, Sabrina Mércia dos Santos Siebra, André de Macêdo Medeiros, Teresinha Silva de Brito, Sonia Elizabeth Lopez Carrillo, Israel Junior Borges do Nascimento, Sidnei Miyoshi Sakamoto, Maiara de Moraes

**Affiliations:** 1,4,5,7 Department of Health Sciences, Biological and Health Sciences Center, Federal University of the Semi-Arid Region, Mossoró, Rio Grande do Norte, Brazil; 2,3,6 Department of Biomedical Sciences, Faculty of Health Sciences, University of the State of Rio Grande do Norte, Mossoró, Rio Grande do Norte, Brazil; 8 University Hospital and School of Medicine, Federal University of Minas Gerais, Belo Horizonte, Minas Gerais, Brazil; 9 Department of Pathology, Health Sciences Center, Federal University of the Rio Grande do Norte, Natal, Rio Grande do Norte, Brazil

**Keywords:** Coronavirus, Confinement, Severe Acute Respiratory Syndrome, Physical Distancing

## Abstract

To provide a synthesis of diverse evidence on the impact of the non-therapeutic preventive measures, specifically quarantine, physical distancing and social isolation, on the control of COVID-19. A scoping review conducted in the PubMed, Embase, LILACS, CENTRAL and SCOPUS databases between 2019 and August 28th, 2020. The descriptors used were the following: “quarantine”, “physical distancing”, “social isolation”, “COVID-19” and “SARS-Cov2”. Studies that addressed the non-therapeutic preventive measures in people exposed to SARs-CoV-2 in community settings and health services were included. A total of 14,442 records identified through a database search were reduced to 346 studies and, after a standardized selection process, a total of 68 articles were selected for analysis. A total of 35 descriptive, cross-sectional or longitudinal observational studies were identified, as well as 3 reviews, in addition to 30 studies with mathematical modeling. The main intervention assessed was social distancing (56.6%), followed by lockdown (25.0%) and quarantine (18.4%). The main evidence analyzed points to the need for rapid responses to reduce the number of infections, deaths and hospital admissions, especially in intensive care unit beds.The current review revealed consistent reports that the quarantine, physical distancing and social isolation are effective strategies to contain spread of the new coronavirus.

## Introduction

On March 11th, 2020, the World Health Organization (WHO) declared the pandemic of the new betacoronavirus of the Coronaviridae family, called Severe Acute Respiratory Syndrome Coronavirus (SARS-CoV-2) and the disease is termed as Coronavirus disease 2019 (COVID-19). Since then, the scientific community worldwide has been trying to discover the potential vaccines and treatments with proven safety and efficacy against the COVID-19 [1,2]. With an estimated pooled basic reproduction number (R0) of 3.32 (95% CI, 2.81 to 3.82) [3], COVID-19 is highly contagious disease which leads to medical emergency and socioeconomic crisis around the world [4].

Transmission rate of direct airborne pathogens, such as SARS-CoV-2, depends on the population density. Therefore, control measures that include the implementation of non-therapeutic preventive measures (NTPMs), such as quarantine, physical distancing and social isolation, play a critical role [5,6]. NTPMs emerged as effective strategies to reduce both infection transmission and the lethality rate. In addition to that, they acted as an attenuating factor of subsequent “waves” during the epidemic, such as the case of the Influenza A (H1N1) pandemic [7-9]. However, previous viral outbreaks did not result in the same behavioral change seen around the world in response to COVID-19, which reinforces the role of these measures in controlling the disease.

Some systematic reviews showed that NTPMs exert a beneficial effect in controlling the spread of respiratory viruses [7,10]. Likewise, Nussbaumer-Streit et al. (2020) [11], suggested that quarantine is important to reduce the incidence of COVID-19 and its associated mortality during the pandemic, if carried out early in time and combined with other public health measures. In addition, physical distancing and social isolation help to minimize the effects of the demand for health care [12,13]. Together, these NTPMs reduce the number of accrued cases, flatten the epidemic curve, and prevent the healthcare system from collapsing.

In this context, it is important to clarify the extension, scope and nature of the diverse evidence available on the NTPMs and their influence on public health. Considering the need for a scientific evidence to support these control measures, the current scoping review methodically examines the literature during the pandemic. Our intention is to produce a synthesis of the peer-reviewed articles that specifically address quarantine, physical distancing and social isolation as NTPMs, as well as to identify gaps and provide the most up-to-date evidence of the usefulness of these strategies in disease control and, in this way, contribute to understanding the impact of these interventions to control COVID-19 disease transmission.

## Methodology

### Protocol and registration

We conducted a scoping review based on the Joanna Briggs Institute methodology [14,15] and on the Preferred Reporting Items for Systematic reviews and Meta-Analyses extension for Scoping Reviews (PRISMA-ScR) [16]. An a priori protocol was projected (https://osf.io/4d6q3/) and registered (Open Science Framework [OSF] ID: DOI 10.17605/OSF.IO/4D6Q3).

### Eligibility criteria

The study included articles published between 2019 and 2020 that investigated individuals (regardless of age, gender, and ethnicity) potentially exposed to the new coronavirus, from countries with the COVID-19 outbreak already declared, in close contact with a confirmed case of the disease or who lived in areas with high transmission rates of SARS-CoV-2 and that explored NTPMs (specifically quarantine, physical distancing and social isolation) in community settings including houses, schools, workplaces and/or healthcare service providers i.e. hospitals, basic health care units, emergency and intensive care units.

### Information sources and research strategy

The research team conducted comprehensive bibliographic searches between December 2019 to August 28th, 2020, in five databases: MEDLINE/PUBMED, CENTRAL, Scopus, LILACS and Embase. Due to the large number of articles identified, those that were not written in English, Portuguese or Spanish were excluded. Quantitative, qualitative and mixed-methods research studies were included. Systematic reviews, text documents and opinion articles were also considered for inclusion. A highly sensitive search strategy for MEDLINE was designed combining clusters of synonyms of keywords and free text search terms for COVID-19, combined with clusters for the NTPMs (see Appendix 1 for the complete search strategy). The search strategy, including all the keywords and MeSH terms identified, was adapted for the other databases.

### Study selection process

After the search, all the records identified were grouped and loaded into a bibliographic management software program called Covidence (Veritas Health Innovation, Melbourne, Australia) [17]. To ensure reliability among the reviewers, a series of training exercises was conducted before the screening. Subsequently, in groups of two independent reviewers were assigned to select the titles and abstracts for inclusion. Using the same process, groups of two reviewers later screened the full text of potentially relevant articles to determine their suitability using similar inclusion and exclusion criteria. Any discrepancy between the reviewers was solved by a third evaluator.

### Data items and data collection process

Data extraction was performed in duplicate and in an independent way using a standardized data extraction table in Microsoft Excel® (Seattle, WA, United States). The data were extracted from texts, tables and graphs. Conflicts were solved by consensual discussions. The data collected were as follows: study design and type, patient's demographics, author(s), publication date, country, study objectives, characteristics of **Data items and data collection process** the population, type of intervention (including its duration and location), comparator, type of outcomes measured, methodology used, conclusions and results related to the outcomes measured.

## Results

### Selection of evidence sources

**Figure 1.**
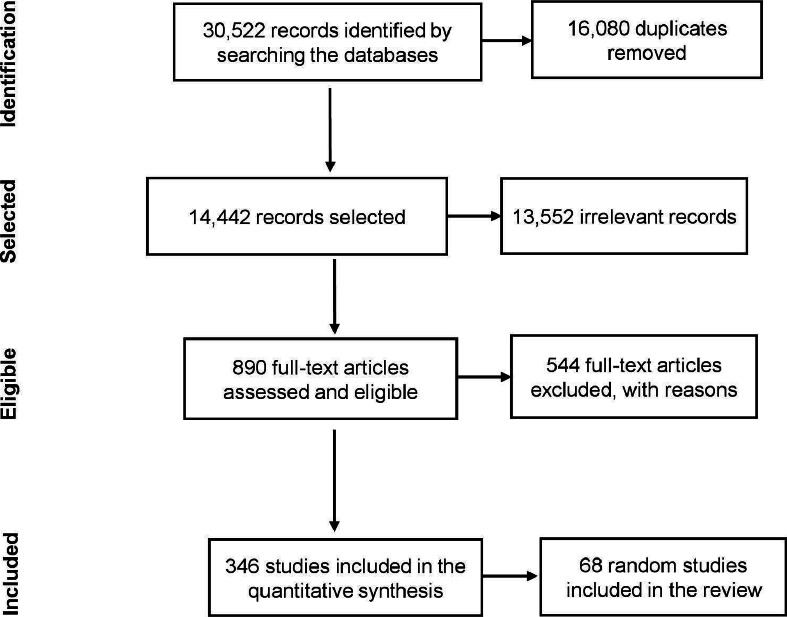
PRISMA flowchart extracted from Covidence® [17] corresponding to the article search and selection stage of the scoping review

After removing duplicates, our search identified 14,442 unique citations, of which 890 articles were evaluated through full-text reading and 346 studies were eligible for inclusion (Figure 1). 544 articles were excluded for various reasons: not meeting the eligibility criteria defined in the scope of the review, descriptive articles without the presence of data (interviews) or that did not specify the impact of the measures, comments, technical notes, errata and articles that were unavailable or in a language outside the inclusion criteria.

The individual characteristics and demographics of the studies with extracted data were described in tables, in addition to a narrative synthesis and mapping of the main results. Due to the large number of eligible articles identified, the data were extracted from 76 articles, selected for convenience in Covidence software, of which 68 were included for analysis after peer review (Table 1), representing 22% of the articles analyzed.

### Characteristics of the evidence sources

#### Countries (distribution)

**Figure 2.**
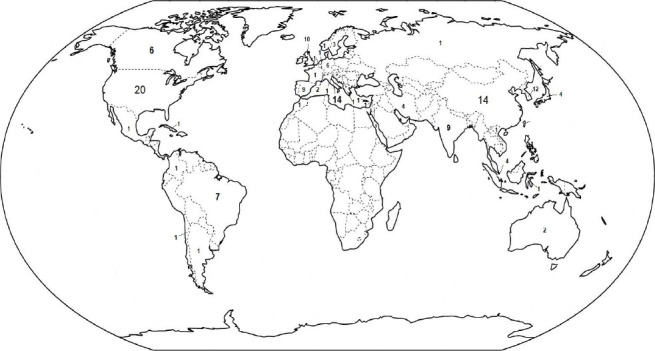
World map with the geographical distribution of the number of papers included in the review according to the countries where the studies were conducted.

Data from at least 36 nations were included and all the continents were represented, as shown in Figure 2.

#### Population

The large majority of the studies assessed the general population, those potentially at risk, asymptomatic cases or already confirmed COVID-19 cases. Others evaluated hospitalized patients or individuals in places such as hotels, military bases, call centers, schools and restaurants.

#### Designs and methodologies of the studies

The methodological approach of the studies reviewed encompassed descriptive, cross-sectional or longitudinal observational studies (n=35; 51.5%). In addition to that, some reviews were also included (n=3; 4.4%). Thirty studies, which represent 44.1% of the total, used mathematical modeling, either in silico models or forecast models through time series (Table 2). Of these, most used the SIR or SEIR models and their adaptations.

### Types of intervention

The interventions include the preventive measures listed in the review: lockdown, quarantine and social/physical distancing (Table 2). More than half of the studies (56.6%) assessed social distancing, proportionally followed by lockdown (25.0%) and by quarantine (18.4%). Most of the analyses performed in the articles resorted to before-and-after comparators of the interventions analyzed, that is, with or without intervention. In some cases, the comparators were separated by levels of interventions and/or scenarios.

#### Descriptive analysis of the main outcomes

In relation to the social distancing measures, Du et al. [18] showed that, in China, there was a 54.3% reduction in the reproduction index 7 days after implementing social distancing. A one-day delay in implementing this measure led to a 2.41-day delay in containment of the pandemic [18]. Bertuzzi et al. [19] (2020) compared the case doubling time in Brazil before and after the implementation of the distancing measures and observed that, before the measures, the case doubling time was 5 days, indicating that, if this trend had continued, the total number of ICU beds needed would have exceeded the capacity within a few days. However, only two weeks after implementing distancing, an increase in the doubling time was observed: from 5 to 18.7 days. This tendency was observed throughout time, with a reduction in the number of occupied ICU beds and an increase in the doubling time to 60.5 days [19].

In the United Kingdom, a study with online questionnaires noticed a 74% reduction in the mean daily number of contacts observed by participant. Most of the contacts (57.6%) occurred at the homes. That was enough to reduce the doubling rate from 2.6 to 0.62 after practicing social distancing. Consequently, behavioral monitoring and the restrictions adopted lead to a reduction in the COVID-19 transmission rate [20]. In the United States, a survey collected information about the efficacy of social distancing in mitigating spread of COVID-19 and the result of the infection rates. Overall, the measures translated from a 12% reduction in the number of cases per day (and therefore 5-6 day doubling) to 5% cases per day and, therefore, 14-day doubling [21]. Another study showed that social distancing was associated with 29% and 35% reductions in incidence and mortality due to COVID-19 [22], respectively. In addition, social distancing reduced COVID-19 transmission in the US by 6.6%, with those with a large population or high population density benefiting the most [23]. In South Korea, only 58 confirmed cases out of a total of 599,000 people (which represents 9.68 cases per 100,000 people) were reported in the Armed Forces until the end of June 2020, which shows a valuable achievement of the policy of social distancing of military people [24]. Another data showed that, with social distancing, the number of new confirmed cases dropped from 37 (19.8%) to 3 (6.1%). In addition to that, the infection rate decreased to less than 10 patients a day and there were days with no new endemic patients [25].

In modeling studies, Brett and Rouhani [26] showed that social distancing measures could lead to the suppression of COVID-19 cases in the United Kingdom, reducing the reproduction rate to less than 1. In Brazil, maintaining the social distancing intervention would make the number of infected people to fall from 2 million to approximately 250,000. If social distancing were put in place for 60 days, the number of deaths would drop from 180,000 (scenario with no distancing) to 140,000, post 100 days of the first reported case [27]. In India, estimates with different restriction levels showed that, with 80% of social distancing, the number of cases reached 4,000 (peak); with 85%, it would drop to 3,000 and, with 90%, to 2,500. The number of estimated cases would increase geometrically if the social distancing norms were relaxed and in-person contact increased [28]. In Iran, when the distancing estimate increased from 25% to 32%, there was a reduction of almost 5,000 deaths, while when the increase was from 10% to 40%, there was a reduction of 300,000 infected cases [29]. Using the city of Seattle (US) as a base and mathematical model, Matrajt et al. [30] showed that the number of hospitalizations and deaths could have been reduced by 78% during the first 100 days after putting the preventive measures into practice. Another North American study estimated a 95% reduction in the transmission rate, in addition to a 64.6% reduction in the hospitalization rate, and a 74.7% reduction in cumulative deaths after adopting the social distancing measures [31]. In Canada, with the social distancing measures, the ICU bed occupancy rate reached 2 per 100,000 inhabitants, with 2.5 deaths per 100,000 inhabitants. Without these measures, the model projected the ICU bed occupancy to be 37.4 per 100,000 inhabitants and 12.7 deaths per 100,000 inhabitants. Consequently, social distancing managed to effectively mitigate spread of the disease [32].

A case report study that evaluated the effects of quarantine in a hotel in the Canary Islands (Spain) showed that PCR tests identified 7 positive individuals for COVID-19, and 6 of them belonged to the group in close contact with the positive patient. Efficacy of this preventive measure was over 99%, since only 7 cases were positive in a population of 1,000 individuals between guests and employees [33].

As for the studies that evaluated the most rigid lockdown, in India, the estimated R value dropped before the first lockdown from 3.36 on March 24th, 2020, to 1.71 on April 14th, 2020. Since then, the estimated R value dropped at a slower pace. The mean R value in the last week of May 2020 was 1.27 [34]. Another study compared lockdown data in three different countries (USA, Italy and Canada) and showed that, in Italy, the initial R (before the first lockdown) was 0.22, corresponding to a DT of 3.15 days. R dropped to 0.1 two weeks after the lockdown, increasing the DT to 7 days. In the USA, R was 0.3 between March 1st and March 21st, 2020 and, in Canada, R rose from 0.13 to 0.25 in two weeks, corresponding to a DT of 2.7 days. The authors pointed out that immediate and far-reaching interventions were needed to counteract the rapid initial growth of the COVID-19 epidemic in Canada [35].

Before the lockdown, the daily percentage increase of all the incidence outcomes was higher in Spain (38.5% for diagnosed cases, 59.3% for deaths and 26.5% for ICU admissions) than in Italy (21.6%, 32.8% and 16.7%, respectively). During the first period of the lockdown measure, both countries showed decrease trends on daily basis (12.5%, 13.7% and 3.7% in Italy; and 11.9%, 17.6% and 9.6% in Spain). Consequently, during the first lockdown, progress of the epidemic was considerably reduced. In Italy, the diagnosed cases decreased 42.1%, deaths dropped 58.2% and ICU admissions were reduced by 77.8%. These reductions were even greater in Spain, where the number of diagnosed cases decreased 69.1%, deaths dropped 77.8% and the ICU admissions were reduced by 66.8%. During the second and more restrictive lockdown, both countries showed some positive signs. Specifically, in Italy, all results started to decline (2% reduction in diagnosed cases, 0.2% reduction in the number of daily deaths and 16.8% reduction in ICU admissions). Similarly, Spain also showed decreasing trends (2.7%, 1.8% and 5.6%, respectively) [36].

In an Italian modeling study, it was estimated that early implementation of the lockdown could have prevented nearly 126,000 COVID-19 cases, 54,700 admissions outside the ICUs, 15,600 ICU admissions and 12,800 deaths, corresponding to 60%, 52%, 48% and 44% reductions, respectively. Therefore, if the lockdown was implemented late in time, there would be an increase in the proportion of hospital admissions and deaths associated with the pandemic [37]. In the United Kingdom, the estimated initial R value was 6.94. In a 12-week lockdown, there was a 5% reduction in the transmission parameters, with peak forecasts of nearly 90,000 severe patients, 25,000 critically ill patients and 44,000 cumulative deaths. With transmission rising from 5% to 30%, 50,000 deaths and 475,000 active cases were expected. The model also forecasted that, if the lockdown had been initiated one week before the actual scenario, nearly 30,000 deaths would have been spared [38]. In India, a number of models pointed out that, before the 3rd phase of the lockdown, the R value was 2.05. Three different periods of the 3rd phase of the lockdown were observed, as follows: from May 4th to May 17th, 2020, with R = 1.6392; from May 18th to May 31st, 2020, presenting R = 1.0245; and from June 1st to August 16th, 2020, with R = 0.4098. It was therefore forecasted that the lockdown would be able to significantly reduce the doubling rate in time. The cumulative number of the population infected was also reduced in this phase of the lockdown, in addition to delaying the appearance of the peak, that is, flattening the contagion curve [39].

## Discussion

In general, NTPMs bring about benefits by reducing the number of new confirmed cases, mortality and basic reproduction rates, as well as the number of hospitalizations, contributing to controlling the pandemic. The outcomes herein analyzed point to the importance of time for the implementation of measures to speed up containment of the pandemic, to increase the doubling time of new cases and, consequently, to reduce the burden on health services, especially ICU beds. Although the NTPMs herein evaluated are considered community mitigation measures recommended during pandemics, questions arise about awareness of the disease diagnosis and the need for behavioral changes. Beckett et al. [40] highlight the increase in social awareness regarding the epidemic associated with social distancing as an important factor in the transmission dynamics, contributing to the plateau and reduction of the epidemic curve. Corroborating this idea, Jarvis et al. [20] show that behavioral monitoring can offer a fast perception of COVID-19 transmission. In this sense, Cowling et al. [41] strongly suggest that social distancing and behavioral changes, which have less social and economic impact when compared to lockdown, can significantly control COVID-19.

Most of the studies analyzed in this review were carried out in locations with high incidence of the disease (such as Lombardy in Italy, in addition to Brazil and the USA), or countries where the influence of population density is an important factor for the progression of COVID-19 (such as India and China). Another important consideration is the issue of age distribution of the disease in the countries made evident by Scarabel et al. [35]. Countries with older populations, such as Italy, tend to suffer more from the pandemic in view of the impact of COVID-19 on this population, with greater severity of cases and a high fatality rate.

Our study identified various mathematical models (Table 1). Some authors [42,43] point out that implementing the lockdown before the beginning of the outbreak is an effective measure to reduce the number of deaths due to the disease. In this sense, Palladino et al. [37] assert that late lockdowns contributed to an increase in the number of deaths and hospitalizations due to COVID-19. The effect of isolation not only reduces the infected population, but also delays the appearance of the epidemic peak [39,43], that is, it saves time for organizing the health system and hospital facilities.

Lockdown is effective where COVID-19 mass testing is large enough to isolate the infected cases [34,44]. For Giordano et al. [43], testing is important because undetected infected people, most of whom are asymptomatic, largely sustain spread of the epidemic. For these authors, the association of a softer lockdown with contact tracking measures and mass testing would strongly contribute to a rapid resolution of the epidemic.

Ibarra Vega [45] highlights that the duration of the quarantine must be cautious, as it can change the contagion curve over time and generate an epidemic peak with a delay time. Homburg [46] also reinforces the effect of delay in decision-making on start of the lockdown, as was the case in Italy, where approximately two-thirds of the total infections with fatal outcomes had already occurred when the lockdown became effective and infections began to decrease. A study conducted in Brazil [19] showed that the early implementation of social distancing and isolation measures reduced the curve of new confirmed positive cases and deaths, corroborating the findings described on the importance of time for the control of the outbreak.

On the other hand, Mahajan and Kaushal [47] showed that, in India, there was a reduction in the number of infected cases, although there was no decrease in mortality, which could reflect the emergence of new epicenters in the country. Another explanation for the changes in trend patterns is that mortality rates require more time to shift in their curves. The delay can also be related to the number of asymptomatic infected people, even with the lockdown measure implemented. In this sense, the time intervals until the results can be identified in the population under study must be considered [48].

According to the study by Ng et al. [49], closing schools does not exert any direct effect on the infection rate; it only delays the epidemic, especially when compared to closing workplaces. In contrast, in a study carried out in the USA, Yehya, Venkataraman and Harhay [50] found diverse evidence that the delayed declaration of the state of emergency and the delayed implementation of school closures were related to higher mortality rates. Park et al. [51] corroborate the idea that the intervention of closing workplaces, such as offices, is effective in containing outbreaks among people in these environments.

McGrail et al. [52] observed a strong reduction in average mobility after the implementation of social distancing policies and indicate a strong correlation between the decrease in the spread of COVID-19 and the reduction in mobility. Corroborating this finding, Siqueira et al. [48] point out that the lockdown is usually accompanied by mobility restrictions, and this could contribute to controlling spread of the disease. The study by Moris and Schizas [53] points out high heterogeneity in terms of the implementation time of the lockdown after the documentation of the first positive COVID-19 case, in addition to indicating that the rapid adoption of quarantine measures, as well as large-scale testing of the population, contributes to a successful outcome. Accordingly, Cowling et al. [41] highlight the role of testing, isolation and tracking of cases, and quarantining close contacts to reduce community transmission of unidentified cases.

### Expert opinion

With the failure of the vaccination process and the ongoing appearance of new SARS-COV-2 variants, the globe has been dealing with a pandemic since 2019 that has the potential to become endemic. It makes sense to adopt sanitary techniques for the epidemiological control of infectious diseases, even if they become endemic.While other techniques of prevention (like vaccines) or treatment (like medications) require time to develop, such approaches should continue to be seen as essential mechanisms. Such measures should continue to be considered indispensable mechanisms while other means of prevention (e.g. vaccines) or treatment (e.g. drugs) take time to be developed.

Physical distancing interventions attempt to prevent the spread of a contagious disease and helps to minimize the impacts on the demand for Intensive Care Unit (ICU) beds, as “staying at home” people contribute to the “flattening” of the virus spread curve, allowing health services to meet the demand for coronavirus-infected patients while also continuing to care for patients with other medical conditions like heart attacks, strokes, and others in ICU beds.

Due to the significant effect and impact of environmental changes, social behavior, and government support to cope with future dangers, especially in the field of zoonotic diseases, infection control techniques should be utilized while we wait for scientific improvement.

## Limitation of the study

We did not train the team in multiple stages to screen the articles, which ended up delaying the screening process, given that doubts arose among the evaluators during the process. We had to review and refine the inclusion and exclusion criteria among peers and, at that moment, we chose to only analyze the articles that explored NTPMs, that is, those that evaluated quarantine, isolation and physical distancing (terminology considered by the authors as the most adequate as a strategy to prevent spread of the virus). Considering the large number of eligible articles identified in the study and the intense and constant number of publications related to this theme, we were not able to perform a good quality analysis of the data from all the articles selected. This would further delay the process to end the review. In this sense, and within our possibilities, we chose to select the papers for convenience herein presented.

## Conclusion

This literature review revealed that the NTPMs (lockdown, quarantine and isolation) contribute to reducing transmission of SARs-CoV-2, as well as to delaying the occurrence of the epidemic curve peak. The main evidence herein analyzed point to the need for rapid responses to contain the COVID-19 pandemic and to reduce the number of infections, deaths and hospital admissions. It is also noteworthy that, although the lockdown, quarantine and isolation of the infected cases are proposed as effective interventions to control the epidemic, they can derive in different results, especially due to the particular characteristics of COVID-19, such as basic reproduction number, cases of asymptomatic infection and occurrence of subclinical infection, in the most diverse regions analyzed and to the different types of study identified.

It is observed that the different approaches used to manage the SARS-COV-2 outbreak indicate the importance of studying the mechanisms by which these measures act under the effects of different contexts, such as cultural and geographic, in addition to the varied economic situations, population density, social interactions, education and social dynamics. Furthermore, systemic factors such as the government, the financial support offered, and even public trust in the government can influence the efficacy of the suppression and mitigation strategies.

Social distancing, confinement, isolation and other public health interventions (case detection with testing and isolation, contact tracing and quarantine) are essential to prevent spread of disease and save lives. Comparing the mechanisms and the various contexts will help policy makers obtain evidence for future pandemics; it will also clarify how to develop and program interventions at the local level for an effective outbreak management.

## Figures and Tables

**Table 1:** Characteristics of the 68 articles randomly selected and included in the scoping review. Own elaboration. Natal/RN. 2021.

MODELING STUDIES				
Author, date	Country	Study objectives	Study design	Intervention
Atangana A, 2020 [54]	Italy	To confirm or discard the effect of the lockdown as an adequate measure to help level out the death and infection curve.	Modeling study/Susceptible - Exposed - Infected - Recovered (SEIR) model/Fractional order COVID-19 model	Lockdown
Beckett *et al*, 2020 [40]	Georgia, United States.	To project the dynamics of COVID-19 spread at the county level in Georgia and to assess the benefits of the interventions, focusing on sustained efforts to reduce infection through different social distancing levels.	Modeling study/Metapopulation AGE-structured epidemiological (MAGE) model	Social distancing
Brett T; Rohani P, 2020 [26]	United Kingdom	To simulate SARS-CoV-2 spread controlled by individual self-isolation and mass social distancing.	Modeling study/Age-structured SEIR model	Social distancing
Brugnago, 2020 [55]	Belgium, Brazil, United Kingdom (UK) and USA	To propose strategies to flatten the power law curves for COVID-19, and to discuss what the effect would be of early, current and late non-pharmacological actions to flatten the curves.	Modeling study/Modified SEIR model	Social distancing
Delen *et al.*,2020 [56]	26 countries from the European Center for Disease Prevention and Cleft	To study the role of social distancing policies in 26 countries and to analyze the COVID-19 transmission rate.	Modeling study/Susceptible- Infected - Recovered (SIR) model	Social distancing
Dickens BL *et al.*, 2020 [57]	Wuhan, China	To compare the impact of two types of isolation: in shelters and at homes	Modeling study	Social distancing
Dropkin G, 2020 [38]	United Kingdom	To predict different lockdown scenarios on parameters related to the COVID-19 pandemic in the United Kingdom.	Modeling study/SEIR model	Lockdown
Gaeta G, 2020 [58]	Northern Italy	To discuss, through comparisons using statistical models, the effects of different strategies such as social isolation, early detection and contact tracking on the dynamics of the epidemic.	Modeling study/SIR model and Asymptomatic infected SIR (A-SIR) model	Social distancing
Gerli AG *et al.*, 2020 [42]	European Union, Switzerland and United Kingdom	To forecast the mortality trends in the 27 European Union (EU) countries, in addition to Switzerland and the United Kingdom, where the lockdown dates and the confinement interventions have been heterogeneous, as well as to explore their determinants.	Modeling study/Multivariate prediction model for individual prognosis or diagnosis	Lockdown
Giordano, 2020 [43]	Italy	To propose a new model that predicts the evolution of epidemics and helps to assess the impact of different strategies to contain spread of the infection, including lockdown and social distancing, as well as testing and contact tracking.	Modeling study/Susceptible - Infected - Diagnosed - Ailing - Recognized - Threatened - Healed - Extinct (SIDARTHE) model	Lockdown and social distancing
Gupta SD; Jain R; Bhatnagar S, 2020 [28]	Rajasthan, India	To develop a mathematical model to forecast the number of cases, progression of the pandemic and its duration, and to relate it to social distancing levels	Modeling study/Susceptible - Exposed - Infected/Asymptomatic - Recovered with Social Distancing (SEIAR-SD) model	Social distancing
Hu Z; Cui Q; Han J; Wang X; Sha WEI; Teng Z, 2020 [59]	Guangdong, China	To explore the effects of population migrations and quarantine strategies on the COVID-19 variations.	Modeling study/SEIR model with effect of the quarantine	Quarantine
Ibarra-Vega D, 2020 [45]	Fictitious data	To simulate and evaluate different quarantine scenarios (long quarantines, double quarantines, smart or combined quarantines) and to verify efficacy in the reduction of contacts and deaths.	Modeling study/Dynamic models obtained through observations of a system	Quarantine and lockdown
Manchein C; Brugnago EL; da Silva RM; Mendes CFO; Beims MW, 2020 [60]	Brazil, China, France, Germany, Italy, Japan, Republic of Kleft, Spain and United States of America (USA)	To analyze the evolution of COVID-19's time series for the following countries: Brazil, China, France, Germany, Italy, Japan, Republic of Korea, Spain and United States of America (USA)	Modeling study/SEIR model	Quarantine
Matrajt L; Leung T, 2020 [30]	Seattle, Washington	To quantify the efficacy of social distancing. To provide estimates for the proportion of cases, hospitalizations and deaths avoided in the short term and to identify the main challenges in assessing the efficacy of these interventions.	Modeling study/Age-structured SEIR model	Social distancing
Morato MM; Bastos SB; Cajueiro DO; Normey-Rico JE, 2020 [27]	Brazil	To investigate the problem of COVID-19's evolution by means of optimal social distancing policies.	Modeling study/Susceptible - Infected - Recovered model with control of the deaths	Social distancing
National Committee on COVID-19 Epidemiology, Ministry of Health and Medical Education [29]	Iran	To forecast the pandemic trend in Iran through the effect of the weather and of the community's behavioral change (isolation level) on the basic reproductive number.	Modeling study/Dynamic model	Social distancing
Ng *et al.*, 2020 [49]	Canada	To estimate the SARS-CoV-2 transmission projections with varied non-pharmacological interventions in Canada.	Modeling study/Agent-based model	Quarantine and social distancing
Palladino R *et al.*, 2020 [37]	Italy	To assess the effects of late lockdown implementation.	Modeling study/Quasi-Poisson linear regression model	Lockdown
Patrikar S; Poojary D; Basannar DR; Faujdar DS; Kunte R, 2020 [61]	India (research data: Italy, South Korea, USA, United Kingdom, Spain, India, Germany, Iran and China)	To synthesize the available data for some of the main countries affected by COVID-19 and to project the COVID-19 estimates for India and the impact of the public health interventions.	Modeling study/Modified SEIR model (R = Recovered + deaths).	Social distancing
Reno C *et al.*, 2020 [62]	Italy (Lombardia and Emilia-Romagna)	To forecast propagation of the infection and its weight on hospitalizations in different social distancing conditions.	Modeling study/Extended SEIR model - susceptible populations (S), exposed (E), asymptomatic infected (A), infected with symptoms (I), hospitalized (H), recovered (R), susceptible in quarantine (Sq) and exposed in quarantine (Eq).	Social distancing
Sardar *et al.*, 2020 [44]	India	To propose a new mathematical model for COVID-19 that incorporates the effect of lockdown (different scenarios). To study the effect of the social distancing measure imposed by the Government on the reduction in the number of notified cases.	Modeling study/SEIR model	Lockdown
Sarkar; Khajanchi; Nieto, 2020 [39]	India	To develop a new mathematical model for the new coronavirus and to assess the consequences of possible policies, incorporating social distancing and lockdown.	Modeling study/Susceptible - Asymptomatic - Recovered - Infected - Isolated infected - Susceptible in quarantine (SARIIqSq) model	Lockdown
Serhani M; Labbardi H, 2020 [63]	Morocco	To build a modified compartmental epidemiological model (SIR) describing transmission of the SARS-CoV-2 virus according to different containment strategies adopted.	Modeling study/Modified SIR model/Susceptible - Infected - Asymptomatic - Quarantined - Recovered - Dead (SIAQRD) model	Quarantine, social distancing and lockdown
Sharifi *et al.*, 2020 [64]	Iran	To estimate the total number of infections, deaths and hospitalizations related to COVID-19 in Iran under different scenarios of physical distancing and isolation.	Modeling study/Extended SEIR model/Transmission model	Social distancing
Shen *et al.*, 2020 [65]	Hubei, China	To assess the impact of metropolitan quarantine on the trend and route of transmission of the SARS-CoV-2 epidemic from January 23^rd^ to April 8^th^, 2020.	Modeling study/Behavioral model	Quarantine
Tuite *et al.*, 2020 [32]	Ontario, Canada	To explore the effect of physical distancing on COVID-19 transmission.	Modeling study/Transmission model	Social distancing
Ullah; Khan, 2020 [66]	Pakistan	To propose a new transmission model that analyzes the dynamics and impact of the non-pharmacological measures against COVID-19 in Pakistan.	Modeling study/Transmission model	Social distancing
Wang X *et al.*, 2020 [31]	Austin, USA	To quantify the life-saving importance of proactively isolating vulnerable populations; we projected the impacts of relaxation with and with no additional measures for vulnerable populations.	Modeling study/Granular model	Social distancing
Wu *et al.*, 2020 [67]	Ontario, Canada	To develop a transmission model taking into account the mitigation strategies implemented in Ontario: physical distancing, contact tracking and diagnosis. To assess transmission risk and the efficacy of the interventions.	Modeling study/Extended SEIR Model - Susceptible (S), exposed (E), asymptomatic infected (A), infected with symptoms (I) and recovered (R), according to the epidemiological panorama, status of the individuals and, subsequently, as diagnosed (D), susceptible in quarantine (Sq), and exposed isolated (Ed) (SEAIRDSqEd)	Social distancing

REVIEW STUDIES				
Author, date	Country	Study objectives	Study design	Intervention
Aquino, 2020 [68]	Germany, Spain, France, Italy, United Kingdom, Sweden, Netherlands, United States, Austria, England, Singapore, India, Brazil	To analyze the impact of the social distancing policies on the COVID-19 pandemic and the challenges for their implementation in Brazil, in order to broaden the population's understanding of their need and provide subsidies for decision-making by managers.	Narrative review	Lockdown, quarantine and social distancing
Nussbaumer-Streit *et al.*, 2020 [11]	United Kingdom, South Korea, Canada, China, Hong Kong, Japan, Singapore, Taiwan	To assess the effects of quarantine in isolation (or in combination with other measures) of individuals who have had contact with confirmed COVID-19 cases, who have traveled from countries with a declared outbreak, or who live in regions with high transmission of the disease.	Rapid review	Quarantine and social distancing
Patiño D *et al.*, 2020 [69]	Wuhan/Hubei and more than 19 countries (Argentina, Australia, Brazil, Canada, Chile, China, Colombia, Cuba, Germany, Iran, Italy, Japan, Mexico, Norway, Russia, South Korea, Spain, United States and United Kingdom)	To rapidly describe the strategies adopted by the countries and their impact on controlling COVID-19.	Rapid review	Social distancing

OTHER TYPES OF STUDY				
Author, date	Country	Study objectives	Study design	Intervention
Basu D *et al.*, 2020 [70]	India	To assess the impact/efficacy of lockdown in four consecutive and adjacent periods of the intervention.	Observational, analytical and cross-sectional	Lockdown
Bertuzzi B *et al.*, 2020 [19]	Porto Alegre, Brazil	To describe the response by the hospitals from the city of Porto Alegre, Brazil, in relation to the COVID-19 pandemic.	Observational and descriptive/Case report	Social distancing
Castillo; Staguhn; Weston-Farber, 2020 [21]	United States	To present data from the states with 'stay at home' requests and to examine the effect of these policies on the increase rate of COVID-19 diagnoses.	Observational, analytical and cross-sectional	Social distancing
Cobb JS; Seale MA, 2020 [23]	United States	To investigate the trends in COVID-19 growth rates in relation to whether or not social isolation (shelter in place [SIP]) is implemented in USA counties.	Observational and analytical/Prospective cohort	Social distancing
Courtemanche *et al.*, 2020 [71]	United States	To estimate the impact of four types of social distancing measures on the growth rates of confirmed COVID-19 cases until April 27^th^, 2020.	Longitudinal and observational	Social distancing
Cowling BJ *et al.*, 2020 [41]	Hong Kong	To quantify the changes in the population's behavior in Hong Kong during the COVID-19 outbreak and to describe the likely impact of these changes and public health measures on COVID-19 transmission.	Observational, analytical and longitudinal	Quarantine and social distancing
Du Z *et al.*, 2020 [18]	China	To estimate the speed with which the social distancing measures contain community transmission in each of the 58 Chinese cities.	Observational, analytical and longitudinal	Social distancing
Gironés-Bredy *et al.*, 2020 [33]	Adeje (Canary Islands, Spain)	To report the quarantine experience implemented in a hotel to contain COVID-19.	Observational and descriptive/Case report type	Quarantine
Homburg, 2020 [46]	United States, South Korea, Germany, Austria, Italy, Spain, Switzerland, Sweden, United Kingdom	To assess the efficacy of lockdown on the mortality rate.	Observational and longitudinal/Time series analysis	Lockdown
Hu Chun-Song, 2020 [72]	Wuhan City, China	To perform a preliminary analysis of the COVID-19 cases during lockdown and the travel restrictions implemented.	Descriptive observational	Lockdown
Imai *et al.*, 2020 [73]	Wuhan (Hubei, China), South Korea, Japan, Hong Kong (Special Aleft Region of China), Singapore, Italy	To assess the efficacy of the non-pharmacological measures to mitigate the COVID-19 numbers.	Observational, analytical and cross-sectional	Social distancing
Jarvis, 2020 [20]	United Kingdom	To describe a research study of contact patterns and compliance with physical distance measures in adults in the United Kingdom. To assess the impact of these measures on control of the epidemic, estimating the doubling index (mean number of secondary cases generated per case).	Observational, analytical and cross-sectional	Social distancing and quarantine
Lau *et al.*, 2020 [74]	China	To assess if strict lockdown measures have the potential to slow down virus spread.	Observational, analytical and cross-sectional	Lockdown and social distancing
Mahajan P; Kaushal J, 2020 [47]	India	To present the epidemiological situation during the Lockdown-1 period (from March 24^th^ to April 14^th^, 2020).	Observational and descriptive/Case report type	Lockdown
Marcus JE *et al.*, 2020 [75]	Texas, United States	To observe the efficacy of the preventive measures in reducing COVID transmission in a military base. To examine the first 7 weeks after initiating the implementation of non-pharmacological interventions in a USA Air Force Military Training base.	Observational and descriptive/Case report type	Quarantine and social distancing
McGrail DJ; Dai J; McAndrews KM; Kalluri R, 2020 [52]	United States and another 74 nations	To assess the efficacy of distancing and to show that the implementation of social distancing policies in USA states has corresponded to a reduction in the COVID-19 spread rates, and that the reduction in the spread rate is proportional to the average change in mobility.	Observational and descriptive/Time series	Social distancing
Moris; Schizas, 2020 [53]	Greece	To assess the efficacy of lockdown in different countries, highlighting the performance of Greek society and authorities.	Observational, analytical and cross-sectional	Lockdown
Oh HS; Woong S, 2020 [24]	South Korea	To describe the response of the South Korea Armed Forces in relation to the COVID-19 pandemic.	Observational and descriptive/Case series	Social distancing and quarantine
Park IN; Yum HK, 2020 [25]	South Korea	To assess the effects of social distancing on mitigating COVID-19 infections.	Longitudinal and observational	Social distancing
Park SY *et al.*, 2020 [51]	Seoul, South Korea	To describe the epidemiology of a COVID-19 outbreak in a call center in South Korea and to assess the effect of the quarantine to limit spread of the disease.	Observational and descriptive/Case report type	Quarantine and social distancing
Pham, 2021[76]	Vietnam	To identify the measures most closely associated with successful SARS-CoV-2 control.	Observational, longitudinal and descriptive	Quarantine
Ryu S *et al.*, 2020 [77]	South Korea	To analyze transmission of the coronavirus disease outside the provincial region of Daegu-Gyeongsangbuk in South Korea.	Cross-sectional and observational	Quarantine and social distancing
Sang WP *et al.*, 2020 [78]	South Korea	To describe potential roles of social distancing in mitigation of COVID-19 by comparing metropolitan traffic data to transmission in 2 main cities.	Cross-sectional and observational	Social distancing
Scarabel F; Pellis L; Bragazzi NL; Wu J, 2020 [35]	Italy, Canada, United States	To forecast the tendency for a COVID-19 outbreak in Canada through data comparison, using Italy as a comparator.	Longitudinal and observational	Lockdown
Siqueira CAS et al., 2020 [48]	Spain	To analyze the impact of the physical distancing measures imposed by the Spanish autonomous communities on incident cases, hospitalizations and mortality trends related to COVID-19.	Observational and analytical/Ecological type	Lockdown and social distancing
Sultanoglu, Baddal; Sanlidag, 2020 [79]	Northern Cyprus	To provide a real-time analysis of the presence of COVID-19 in northern Cyprus.	Cross-sectional and observational	Quarantine
Tobias A, 2020 [36]	Italy and Spain	To describe, quantify and compare the effects of lockdown within and across countries from the epidemiological point of view using incidence data.	Observational, descriptive and cross-sectional	Lockdown
Vaishnav; Vajpai, 2020 [80]	India	To analyze the effect of opening or relaxing the lockdown on the spread of coronavirus in India and to predict its national quantitative spread for the next six months.	Observational and longitudinal/Time series	Lockdown
Valentine R; Valentine D; Valentine JL, 2020 [81]	United States	To examine the impact caused by the demonstrations and breach of social distancing on the COVID-19 infection rate.	Observational and longitudinal - Event study methodology	Social distancing
Vopham *et al.*, 2020 [22]	United States	To examine the associations between State policies and social distancing measures, as well as between social distancing and incidence and mortality due to COVID-19.	Observational, analytical and cross-sectional	Social distancing
Wagner AB *et al.*, 2020 [82]	United States	To analyze, test and quantify the efficacy of social distancing, adequately correcting the delays in its effect.	Observational and longitudinal/Time series	Social distancing
Wee LE *et al.*, 2020 [83]	Singapore, Asia	To observe the result implementing social distancing measures employed at the Singapore General Hospital during the COVID-19 outbreak (in-hospital transmission).	Longitudinal and observational	Social distancing
Yehya NY, Venkataramani A; Harhay MO, 2020 [50]	United States	To assess the association between the delay in the emergency declarations and in school closures and the subsequent mortality due to COVID-19.	Observational and analytical/Ecological type	Social distancing
Zhao *et al.*, 2020 [84]	Wuhan, China	To estimate transmissibility of COVID-19 and to explore the relationship between the instantaneous basic reproduction number (R0t), public awareness and the effect of the lockdown strategy in Wuhan.	Observational and analytical/Baidu Index and Baidu Migration Scale (BMS)	Lockdown
Zobbi MAL *et al.*, 2020 [85]	Australia, Jordan, United States, Indonesia, Sweden, Brazil, Italy, Lebanon, India, Germany, South Korea, Spain	To highlight some factors of lockdown that can exert a direct impact on the contagion level. To assess the efficacy of lockdown.	Observational and longitudinal/Statistical model	Lockdown

**Table 2.** Frequency (%) of the types of study designs and interventions of the articles included in the scoping review. Own elaboration. Natal/RN. 2021.

		Frequency (%)
Study design	Mathematical model	44.1
	Observational study	51.5
	Review	4.4
Interventions	Social distancing	56.6
	Lockdown	25.0
	Quarantine	18.4

## Appendix

**Apendix 1.** Search strategies

Search*	Query	Recovered records
#1	(((((("Coronavirus"[MeSH] OR "Coronaviruses") OR ("Coronavirus Infections"[MeSH] OR "Coronavirus Infection" OR "Infection, Coronavirus" OR "Infections, Coronavirus")) OR ("Betacoronavirus"[MeSH] OR "Betacoronaviruses")) OR ("severe acute respiratory syndrome coronavirus 2"[Supplementary Concept] OR "2019-nCoV" OR "Wuhan coronavirus" OR "2019 novel coronavirus" OR "COVID-19 virus" OR "coronavirus disease 2019 virus" OR "COVID19 virus" OR "Wuhan seafood market pneumonia virus")) OR ("COVID-19"[Supplementary Concept] OR "2019 novel coronavirus disease" OR "COVID19" OR "COVID-19 pandemic" OR "SARS-CoV-2 Infection" OR "COVID-19 virus disease" OR "2019 novel coronavirus infection" OR "2019-nCoV infection" OR "coronavirus disease 2019" OR "coronavirus disease-19" OR "2019-nCoV disease")) OR ("Respiratory Distress Syndrome, Adult"[MeSH] OR "ARDS, Human" OR "ARDSs, Human" OR "Respiratory Distress Syndrome, Acute" OR "Acute Respiratory Distress Syndrome" OR "Adult Respiratory Distress Syndrome")) OR ("Severe Acute Respiratory Syndrome"[MeSH] OR "Respiratory Syndrome, Severe Acute" OR "SARS (Severe Acute Respiratory Syndrome)" OR "Respiratory Syndrome, Acute, Severe")	78,695 results
#2	((((("Social Isolation"[MeSH] OR "Isolation, Social" OR "Isolations, Social" OR "Social Isolations") OR ("Quarantine"[MeSH] OR "Quarantines")) OR ("Infection Control"[MeSH] OR "Control, Infection")) OR ("Physical Distancing" OR "Social Distancing" OR "social distance")) OR ("Patient Isolation")) OR ("Lockdown")	87,969 results
#3	#1 AND #2	3,370 results
Limited to publication date on 2019-2020		2,603 results
